# Commentary: The Expression of CD123 Can Decrease with Basophil Activation: Implications for the Gating Strategy of the Basophil Activation Test

**DOI:** 10.3389/fimmu.2016.00260

**Published:** 2016-07-07

**Authors:** Salvatore Chirumbolo

**Affiliations:** ^1^Department of Neuroscience, Biomedicine and Movement-University of Verona, Verona, Italy

**Keywords:** basophils, CD123, CD203c, bias, flow cytometry

Basophil activation test (BAT) is an assay used to diagnose allergy by evaluating the expression of surface molecules in circulating and activated basophils with an *in vitro* flow cytometry (FC) approach. Separation of basophils from whole blood is virtually made by an electronic capture in FC. This process is called gating and uses a “phenotyping” protocol that targets membrane molecules typically or exclusively expressed by circulating basophils. Once “gated,” basophils are examined for their expression of molecules, such as CD63, CD203c, or CD193 (CCR3), usually up or downregulated by cellular activation from allergens or non-IgE-mediated stimuli.

A recent article by Santos et al. reported that the CD123/HLA-DR protocol used to gate basophils in an FC approach could be fully improved by considering the introduction of CD203c as a gating additional molecule, as they reported a degree of correlation between basophil activation (as measured by the increased expression of CD203c) and decreased expression of CD123 without making a direct causal link between both the observations (1). They actually showed that, during cellular activation, the expression of membrane CD123, the alpha subunit of the interleukin-3 (IL-3) receptor, is downregulated, leading to a possible loss in the electronic capture of basophils and hence a false negative outcome of the activation test (1). Moreover, they suggested the introduction of CD203c marker, quite exclusively expressed by basophils, to improve BAT performance (1). In their paper, the authors hypothesized also that activated basophils should upregulate membrane expression of the class II MHC antigen HLA-DR, but their results confirmed that basophils do not express HLA-DR, even during allergic activation (1–4).

The paper by Santos et al. contains interesting issues, which raised comments from this author.

This evidence represents a novelty in the field, as it reports that a phenotyping marker, i.e., CD123, should be downregulated during basophil activation. In this regard, the evidence by Santos et al. would suggest that gating strategies using CCR3 (CD193), the eotaxin receptor, may be revised, as CCR3 is notoriously downregulated during basophil activation and may therefore behave as CD123, leading to considerations that would be very interesting to address in next future (1, 3, 5, 6). To the best of our knowledge, CD123 has never been considered an activation marker (7). Apparently, its association with a basophil activation marker, exclusively expressed by basophils, such as CD203c, should improve the ability to capture or gate these cells in FC (1, 7).

The phenotyping protocol widely used to gate basophils includes both CD123 and HLA-DR (4). Santos et al. reported that while basophil HLA-DR remains unexpressed during any kind of activation, either IgE- or non-IgE-mediated, CD123 is downregulated on basophil membrane, then it led to a reduction in CD123-fluorescent cells captured as dots events in the FC analysis and to possible false negatives in the BAT evaluation (1). Previous reports of our group showed that CD123 is highly expressed (“bright”) on the membrane of basophils undergoing an *in vitro* BAT *prior* of any activation (3, 4). We also demonstrated, by evaluating the mean fluorescence (MFI_CD123_) in the gate, that the behavior of CD123, in the protocol CD123^bright^/HLA-DR^neg^ used to gate basophils, was unchanged even following different kinds of activation, i.e., CD123 did not significantly change its membrane expression following either an IgE- or a non-IgE-mediated basophil activation (3, 4); and furthermore, no evidence of direct inhibition of CD123 following basophil activation has been ever reported in the literature. My modest opinion is that the observed apparent reduction of CD123-FITC may be caused by effects related to the gating strategy involving CD203c.

If we gate basophils through a SSC/CD203c approach and then evaluate the expression of CD123 and HLA-DR, we might be biased about the expression of CD123 by the fact that CD203c changes its fluorescence pattern upon activation. Yet, the opposite approach, i.e., gating with a CD123/HLA-DR and then with CD203c, was suggested with the purpose to improve basophil capture. A gating protocol adopting CD203c as a phenotyping marker, due to the upregulation of the latter upon activation, may lead to an overestimation of basophil count in the gate.

The authors demonstrated that the introduction of CD203c in the CD123/HLA-DR phenotyping protocol leads to a reduction in the loss of basophils in the gate and ameliorated the ability to catch basophils expressing CD63, thus preventing possible bias in the diagnostic value of BAT (1). The authors showed in Figure 5b of their manuscript, an evidence that should recall perfectly a circumstance published by ours elsewhere [Figure 4, page 8/14 of Ref. (4)], showing the expression of CD63 and CD203c following an fMLP-induced activation, i.e., dot plot of Ref. (1) is quite perfectly akin dot plot of Ref. (4). The authors reported this evidence as particularly meaningful of their conclusions. They assessed that following fMLP activation, the reduction in the number of gated cells with a CD123/HLA-DR protocol was highly significant (*p* < 0.004) compared with anti-IgE (*p* < 0.99), which was exactly the opposite opinion of others, reporting a CD123 quite unchanged upon fMLP activation (1, 4). Interestingly, the image reported in Ref. (4) is perfectly comparable to the one reported by the authors in Figure 5b of their paper and, as Chirumbolo et al. sustained the evidence that CD123 was expressed in a bright form and unchanged during activation (3, 4), the occurrence of the same evidence with opposite opinions on CD123 is particularly intriguing and deserves further research considerations. Moreover, the authors succeeded in counting more than 5,000 basophils in the gate using the same volume of sample. Usually, the theoretical number of basophils in 100 μl heparin-anticoagulated whole blood ranges from 10^3^ to 10^4^, but this number may be much lower due to yield concerns. During activation, the authors reported higher values of basophils in the gate when captured with only CD203c, i.e., SSC/CD203c, showing a slight overestimation due to changes in CD203c membrane expression, although it is possible to argue that the number of basophils in the gate may be underestimated in non-stimulated conditions since low expressing CD203c basophils would be missed.

Aside for the need to expand the debate about improvements in BAT strategy, this analytical test remains a formidable tool to diagnose allergy, and the ability to test activation depends also on the optimization of the gating strategy (8, 9).

## Author Contributions

SC planned the paper and the experimental data, elaborated the results, conceived and discussed the paper, wrote the manuscript, and submitted it to the journal.

## Conflict of Interest Statement

The author declares that the research was conducted in the absence of any commercial or financial relationships that could be construed as a potential conflict of interest.
